# Effects and Treatmentment of Salivary and Mucous Deposits

**Published:** 1870-04

**Authors:** Louis Augspath


### ARTICLE III.

### *Effects and Treatment of Salivary and Mucus Deposits.*

By Louis Augspath, D. D. S.

There is, doubtless, in the whole range of causes nothing which exerts a greater influence upon the profession of dentistry than the neglect, on the part of the mass of mankind, of proper care of the teeth and mouth.

A want of care is beyond all question, the source of most of the diseases and evils to which the teeth are subject. There is, probably, nothing which more properly claims the attention of the intelligent dentist than the subject of deposits, including the calcareous formation usually denominated *Tartar, and the Green and Brown Stains*, and all those impurities on the teeth which are produced by neglect, tobacco and other similar causes.

It is well understood that there are different varieties of tartar, characterized by color, composition and consistency, but all produced by the same cause and resulting as a precipitate of the saliva, in connection, possibly, with deposits of the mucus.

Persons of all ages are subject to deposits of tartar, although it seldom appears before children have erupted their six year molars, but continues to be formed throughout life; and often to such a degree, that teeth may be found nearly if not entirely covered with it, especially in persons who have been repeatedly and severely salivated, or are of a dyspeptic or scrofulous diathesis. In some persons tartar is deposited throughout life, while others are exempt until some constitutional change takes place when it is rapidly eliminated. This deposite in its direct action on the tooth, in regard to health, is innocent, as it is an exterior formation on the surface of the tooth, and serves rather to prevent than to produce decay. On the other hand it is well worth the notice of the dentist and should never be allowed to remain, as from its tendency to increase on the most protected points, it will naturally force the gum to recede, the alveolar process to absorb (where there is pressure there is absorption), and if left unmolested will not only loosen the teeth but finally cause them to drop from their sockets.

Green and brown stains, doubtless, are caused exclusively by the mucus. This stain is not, like tartar, a formation on the tooth, but enters into the composition of the enamel and tends to produce decay and the destruction of the entire tooth. To this disorder young persons are especially liable, as the enamel is of a lower order of density and the acids of the mouth will therefore act upon it with greater rapidity than in more advanced age.

As a general rule the anterior superior incisors are most liable to be attacked by this disease, owing to their position in the dental arch, where the saliva is only sparingly retained, and where the cleansing if not polishing action of the tongue is almost entirely excluded. This will to some extent account for the reason this disease selects the labial surface in preference to any other.

The remedy for the former of these diseases (tartar) is purely mechanical, but for the latter (stain) it may be necessary to combine the mechanical with therapeutic treatment.

Giving attention first to tartar, I shall endeavor to explain the modus operandi in relieving the teeth of these disagreeable and destructive affections. There are two methods of removing salivary calculus from the teeth: the one by mechanically decomposing the deposit by the use of some acid, the other, mechanical, by scaling and scraping with appropriate instruments. The former should never be resorted to, as the chemical action of the acid does not stop with the decomposition of the calcareous deposit, but by the same affinity attacks the tooth itself, and with almost equal readiness destroys it. The removal of tartar by the second method does not involve a very great amount of skill, and with suitable instruments is easily performed. To accomplish the operation with success, appliances and instruments of various forms and curves are necessary, adapted and adjusted to the various situations to be operated on. All instruments should be very sharp; but, in my opinion, with the cutting edge slightly removed. The blade of the instrument should be applied at a slight obtuse angle with the tooth, beyond the edge of the deposite next to the gum, and passing under the tartar thus scale it off to the point of the tooth, in such a manner as not to roughen or in any manner abrade the enamel. Tartar which is deposited on proximal surfaces of the teeth is to be carefully noticed and removed with instruments having very thin blades. After the thick deposits have been removed the surface should then be carefully and gently scraped,so as to thoroughly clean off every particle of the tartar, and afterwards fully and completely polished with fine pumice or arkansas stone, and finished by burnishing. The manipulation of removing tartar is one of the most simple in dental practice, but to be successful in this, as in every other operation, the process should in every instance be performed with the most perfect thoroughness, as neglect or carelessness on the part of the operator will cause a new deposit on a rough surface with great rapidity. In fact a careless operation will often leave the mouth in a worse condition than before the teeth were operated on.

The removal of salivary calculus is perhaps the most unpleasant duty the Dentist is called upon to perform, as the majority of the cases which require it are very disagreeable, and many are positively disgusting. The popular mind seems to be lamentably ignorant on the subject of proper care of the teeth; and it should ever be the duty of the Dentist to inform his patients of the importance of cleanliness, as many are very prone to neglect the matter, either on account of the unpleasantness of the operation, or from ignorance of the necessity of it.

The eradication of *Green* or *Brown Stains* requires some practice—judgement, and a more skilful manipulation than the removal of salivary deposits. As this disease presents itself in three distinct stages I shall speak of the remedies suitable to each one.

Stage I. Where the erosion is but slight, friction with a piece of hard and fine grained wood (such as orange wood) combined with fine pulverized pumice stone, will be found sufficient to correct this evil. The principal seat of the stain being on the neck of the tooth and in close approximation with the free margin of the gum, care should, in every instance, be taken not to wound the soft tissue, such accident, although of no material consequences will have great influence upon the patient. In most cases the operation will ever afterwards be dreaded and most certainly will they complain of the roughness or the unskillfulness of the operator.

STAGE II. The disease having made great progress we will not only find the enamel discolored to a greater extent, but will also find that the disease has carried its ravages to a great depth. In most cases the dentine will be more or less involved; this being the case the tooth, as a general rule, is extremely sensitive to the touch. The disease presenting itself as above described, the enamel chisel and file will be the most appropriate instruments to perform the operation. The chisel, the first instrument brought into service, should be of fine quality, of excellent temper, decided sharpness and well adapted to the surface to be operated on. And here I wish to remark, that all of the above qualities are combined in the instruments known as Dr. B. F. Arrington's enamel chisels. These instruments may be approximated but not surpassed.

Grasping the chisel firmly, and in such a manner as to leave the thumb independent of the movements of the hand, this (the thumb) should rest on a neighboring tooth, in order that the operator may have perfect control over his instrument and avoid the slipping of the same, by which accident the soft tissue would be wounded. This precaution observed, the operator will proceed with a steady and decided movement of his hand, cutting from the edge of the tooth towards the gum, and thus separate the diseased from the healthy tissue.

In all cases the operation will be painful, but in many intolerable; for such, the writer has applied nit. argent (chrystalized) by slightly touching the sensitive dentine, and with the most happy results.

The chisel following each application of the caustic, the diseased tissue will be removed without much inconvenience to the patient, and before the caustic has time to discolor the dentine.

As a precaution against discoloration, it is advisable to apply a neutralizing agent, such as common salt.

By using the chisel carefully the use of file may be omitted, and I prefer to dispense with this latter instrument as the friction produced by it gives unnecessary pain, and does not aid any in the speedy accomplishment of the operation. Having thoroughly removed the diseased tissue the surface is now ready for final finishing, the process being the same as already described in the removal of salivary deposits. Of course, the above treatment is only advisable where the dentine is well calcified and of sufficient thickness to protect the pulp.

Stage III. In this stage of the disease, the dissolution of the dentine will be in very close approximation with the pulp, and in many instances this organ will be found exposed. The treatment applied in the second stage is here not admissible, and should therefor not be attempted, as it would remove too much of the healthy tissue to leave sufficient protection for the vital part, saying nothing of the disfigurement and the weak condition in which the tooth would be left. Under these circumstances our only treatment is to form a cavity of proper shape and to fill accordingly.

In the treatment of the teeth, as with all other diseases physical or moral, there is much truth in the old maxim—"an ounce of prevention is better than a pound of cure." The most potent of all preventives of diseases of the teeth and mouth is cleanliness. Therefore, the dentist should avail himself of every opportunity and neglect no means of impressing upon the minds of his patients their duty in this respect.

Local treatment will many times prove insufficient, even in cases which are not complicated with constitutional diseases, as syphilis, scrofula and the like, therefore every dentist should qualify himself to administer this treatment in his own person, rather than refer his patient to the practitioner of medicine.

This is an imperative duty if we would uphold and support the true dignity of our profession, and demonstrate to the world the validity of our claim to be considered members of an alleviating and healing profession.

